# Transactions of the American Dental Association

**Published:** 1879-06

**Authors:** 


### BIBLIOGRAPHICAL.

*Transactions of the American Dental Association* at the Eighteenth Annual Meeting held at Niagara Falls, August, 1878. Publication Committee: Drs. George H. Cushing, J. N. Crouse and George W. Keely.

This volume contains many interesting essays from the members of the different Standing Committees, and is a valuable addition to dental literature.

